# Vacancies, disorder-induced smearing of the electronic structure, and its implications for the superconductivity of anti-perovskite MgC_0.93_Ni_2.85_

**DOI:** 10.1038/s41598-017-09997-2

**Published:** 2017-08-31

**Authors:** David Ernsting, David Billington, Thomas E. Millichamp, Rebecca A. Edwards, Hazel A. Sparkes, Nikolai D. Zhigadlo, Sean R. Giblin, Jonathan W. Taylor, Jonathan A. Duffy, Stephen B. Dugdale

**Affiliations:** 1 H.H. Wills Physics Laboratory, University of Bristol, Tyndall Avenue, Bristol, BS8 1TL United Kingdom; 2 Japan Synchrotron Radiation Research Institute, SPring-8, Sayo, 679-5198 Japan; 3 School of Chemistry, University of Bristol, Cantock’s Close, Bristol, BS8 1TS United Kingdom; 4 Department of Chemistry and Biochemistry, Freiestrasse 3, University of Bern, Bern, Switzerland; 5 School of Physics and Astronomy, Cardiff University, Queen’s Building, The Parade, Cardiff, CF24 3AA United Kingdom; 6 DMSC - European Spallation Source, Universitetsparken 1, Copenhagen, 2100 Denmark; 7 Department of Physics, University of Warwick, Coventry, CV4 7AL United Kingdom

## Abstract

The anti-perovskite superconductor MgC_0.93_Ni_2.85_ was studied using high-resolution x-ray Compton scattering combined with electronic structure calculations. Compton scattering measurements were used to determine experimentally a Fermi surface that showed good agreement with that of our supercell calculations, establishing the presence of the predicted hole and electron Fermi surface sheets. Our calculations indicate that the Fermi surface is smeared by the disorder due to the presence of vacancies on the C and Ni sites, but does not drastically change shape. The 20% reduction in the Fermi level density-of-states would lead to a significant (~70%) suppression of the superconducting *T*_*c*_ for pair-forming electron-phonon coupling. However, we ascribe the observed much smaller *T*_*c*_ reduction at our composition (compared to the stoichiometric compound) to the suppression of pair-breaking spin fluctuations.

## Introduction

MgCNi_3_ has been the focus of a great deal of interest since the discovery of superconductivity below a critical temperature of *T*_*c*_ ≈ 8 K^1^. The primary cause of this interest was that superconductivity existed at all in the presence of such a high proportion of Ni, suggesting that the compound may be on the verge of magnetic order and harbour strong ferromagnetic spin fluctuations which are antagonistic to spin-singlet superconductivity. This resulted in speculation that MgCNi_3_ might be an unconventional electron-paramagnon superconductor, its pairing driven by interactions involving ferromagnetic spin fluctuations, as speculated for the putative spin-triplet *p*-wave superconductor, Sr_2_RuO_4_^2^. Further interest in this intermetallic compound has stemmed from the fact that it crystallises in the cubic perovskite structure that is the building block of both Sr_2_RuO_4_ and the layered cuprate high-temperature superconductors^3^. The structural similarity that it shares with these compounds led to speculation that it may provide the link between the unconventional superconductivity exhibited by high-temperature superconductors, and conventional superconductivity in intermetallic compounds^4^.

Since the initial surge of interest, there followed significant controversy regarding the nature of the pairing responsible for the superconductivity, with various studies suggesting a possible spin-fluctuation-driven mechanism^5–7^, multiband superconductivity^8^, conventional electron-phonon coupling^1, 9–13^ including a C isotope effect^4^, and some even providing evidence in support of both^14, 15^. The first calculations of the electron-phonon coupling constant, *λ*, indicated that it was relatively large (Dugdale and Jarlborg calculated *λ* ≈ 0.9^16^, and Singh and Mazin suggested that low-frequency rotational modes of the Ni octahedra could contribute a *λ* of about 3 on the lighter FS sheet^17^). Subsequent calculations indicated that the overall *λ* could be as large as 1.51 due to anharmonic effects^12^. The Sommerfeld parameter is also consistent with a *λ* of 1.45, and the jump in the specific heat at the superconducting transition was found to be strongly enhanced compared to the BCS value, and interpreted as being due to strong electron-phonon coupling by Wälte *et al*.^13^. Furthermore, this strong-coupling version of superconductivity is supported by more recent experiments^18, 19^ and theory^20^. Contrary to this, the most recent study, based upon London penetration depth measurements that probe the underlying superconducting gap structure, concluded that MgCNi_3_ is a conventional, *s*-wave, weak-coupling superconductor^21^. However, their observations are also consistent with the antagonistic coexistence of relatively strong pair-forming electron-phonon coupling and pair-breaking electron-paramagnon coupling due to the strong spin fluctuations expected in a material so close to ferromagnetism^22^, the combination of which would conspire to suppress the critical temperature^23^. Further clarification of the nature of the superconductivity requires detailed experimental studies of the electronic structure.

The only direct electronic structure studies have been via x-ray emission, electron photoemission, and x-ray absorption spectroscopy experiments^24, 25^, which probe the total and partial density-of-states (DOS). The samples studied in those experiments were polycrystalline, some of which were slightly deficient in C or doped with Co, with the highest *T*_*c*_ observed close to perfect stoichiometry^26^. Nevertheless, these experiments were consistent with the calculated electronic structure and, significantly, verified the existence of a peak in the DOS just below the Fermi energy, *E*_F_. The height of this peak was, however, found to be greatly suppressed with respect to the existing predictions^5, 16, 17, 24, 27^. Clearly an experimental measurement of the Fermi surface of MgCNi_3_ is highly desirable^28^, and the recent availability of suitable single crystals has made this possible. Here, we have utilised high-resolution x-ray Compton scattering to determine the bulk Fermi surface and electronic structure of a C- and Ni-deficient single crystal with composition MgC_0.93_Ni_2.85_. Compton scattering is not restricted by short electronic mean-free-paths, does not rely on a cleanly cleaved surface, and probes the bulk of the material^29^. These measurements are compared with state-of-the-art electronic structure calculations to help interpret the data and understand the effect of disorder-induced smearing of the quasiparticle states on the bulk electronic structure.

## Results and Discussion

The stoichiometry of the samples was determined by x-ray diffraction (XRD) on one of the smaller single crystals in the growth batch, and found to be Mg_1.00±0.01_C_0.93±0.01_Ni_2.85±0.03_. Samples in this batch typically had *T*_*c*_ ≈ 6.5 K.

In order to understand the experimental results and anticipate the effects of disorder from the presence of vacancies, we first reproduce what is known from earlier calculations about the electronic structure of the stoichiometric compound, before moving on to that of the non-stoichiometric compound. This will provide a basis for understanding the electronic structure of MgC_0.93_Ni_2.85_relative to MgCNi_3_.

### Stoichiometric electronic structure calculations

There is consensus among previous electronic structure calculations about the electronic structure of stoichiometric MgCNi_3_; there is a large peak in the DOS situated just below *E*_F_, comprised of mainly Ni 3*d* and C 2*p* states, that results from a van Hove singularity (vHs) caused by a very flat, high-mass band around the M-points of the simple cubic Brillouin zone^5, 16, 17, 24, 27, 30^. Both this high-mass band (band 1) and a second, much lighter band (band 2) cross *E*_F_, giving rise to hole and electron Fermi surface sheets, respectively. As the unit cell has an even number of electrons, MgCNi_3_ is a compensated metal and these sheets contain equal numbers of holes and electrons. The calculated electronic structure of the stoichiometric compound was able to reproduce the experimentally observed temperature dependence of the Hall coefficient^31^, normal state resistance^32^, and thermopower^33^. From this electronic structure, Wälte *et al*. decomposed the electron-phonon coupling between the hole and electron FS sheets, giving *λ*_1_ ≈ 1.74–1.78 and *λ*_2_ ≈ 2.20–2.42, respectively^13^.

**Figure 1:**
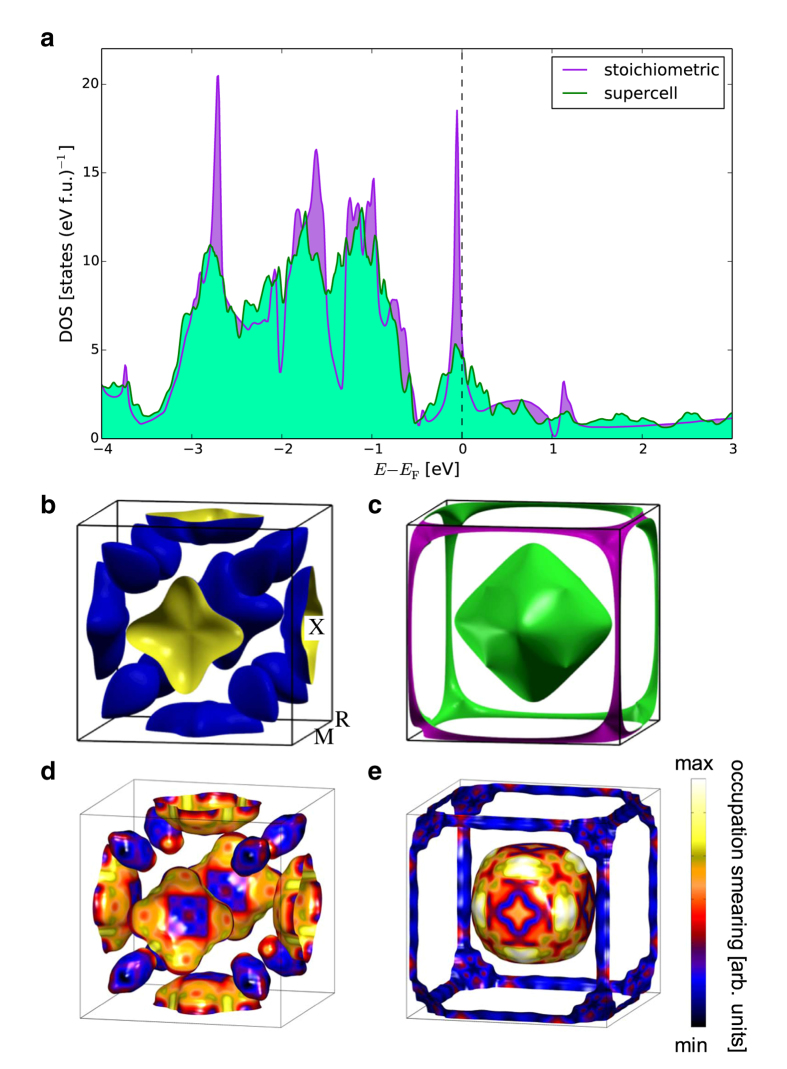
Calculated electronic structure of stoichiometric and non-stoichiometric MgCNi_3_. (**a)**, Comparison of the total DOS for the stoichiometric (purple) and supercell configurational average (green) calculations. Calculated stoichiometric Fermi surface from bands 1 and 2 are shown in (**b** and **c**), respectively. Calculated MgC_0.875_Ni_2.875_ supercell configurationally averaged Fermi surface of bands 1 and 2 are shown in (**d** and **e**), respectively. The colours on the surfaces represent the occupation smearing of the configurational average, and are given by the standard deviation of the occupation density at the Fermi surface. The high-symmetry points of the simple cubic Brillouin zone are labelled in (**b**).

The electronic structure of stoichiometric MgCNi_3_ calculated in the present study is essentially the same as the previous studies: Fig. 1a, b and c show the DOS and Fermi surface sheets, respectively. The DOS at the Fermi energy, *N*(*E*_F_) = 5.63 states (eV f.u.)^−1^, is in good agreement with the previous calculations, and is dominated by band 1, which contributes 83% of *N*(*E*_F_). As in the previous studies, two bands cross *E*_F_ creating a hole sheet (band 1) and an electron sheet (band 2). The band 1 hole sheet consists of an X-centred shell-like feature and eight ovoids located between the Γ- and R-points, whilst the electron sheet from band 2 consists of a Γ-centred distorted octahedron and a jungle-gym connecting the R- and M-points (around the edges of the simple cubic Brillouin zone).

### Non-stoichiometric electronic structure calculations

Calculations for the non-stoichiometric compound were performed with various methods (see *Methods*) in order to attempt to treat the effect of disorder on the electronic structure.

Figure 1a also shows the calculated DOS for a 2 × 2 × 2 supercell configurational average (with one C and one Ni atom removed to give an effective stoichiometry of MgC_0.875_Ni_2.875_). Evaluating the momentum density from such a supercell is already computationally demanding, and obtaining a configurational average from a series of larger supercells was not practical. However, the DOS from a 3 × 3 × 3 supercell was found to be very similar to, and slightly smoother than the smaller supercell, as might be anticipated. The supercell configurational average predicts a very similar DOS (particularly near *E*_F_), but the large peak just below *E*_F_ exhibits a broadening and a significant height reduction, presumably due to a smearing of the vHs in comparison to the stoichiometric calculation. Interestingly, these calculations show no clear shift of *E*_F_ relative to the band manifold (as is predicted by a so-called ‘virtual crystal approximation’ (VCA)^34^ calculation), in agreement with the electronic structure observed by the spectroscopy experiments on polycrystals with C vacancies^24, 25^, and previously predicted to appear in ‘coherent potential approximation’ (CPA) calculations with C vacancies^10^. Because of the proximity of the Fermi level to the diminished vHs, *N*(*E*_F_) is reduced to 4.52 states (eV f.u.)^−1^. This reduction in *N*(*E*_F_) compared with the stoichiometric compound would indicate a reduced propensity for magnetism and spin fluctuations in the presence of C and Ni vacancies (in accordance with the lack of evidence reported for spin fluctuations in experiments performed upon single crystals^19^, compared to near stoichiometric polycrystals^13^). The reduction in *N*(*E*_F_) may also explain why single crystals with C and Ni vacancies are observed to have a lower *T*_*c*_ than polycrystals with small amounts of C deficiency^1, 21, 26, 35^.

The configurationally averaged supercell Fermi surfaces are shown in Fig. 1d and e. The supercell calculations predict the same number of Fermi surface sheets as in the stoichiometric compound, and they retain the same general shape. Although there is no longer an even number of electrons per unit cell, the hole and electron sheets have very similar volumes, as may be expected from the unusual temperature dependence of the Hall coefficient and thermopower (measured in polycrystals)^31, 33^, explained by hole and electron sheets with almost equal volumes^17, 32^. The colours shown on the surfaces represent one standard deviation of the occupation densities from the individual supercell calculations relative to the average, at the configurationally averaged Fermi surface. This provides an indication of the size of the disorder smearing in ***k***-space, and suggests that the Γ-centred electron and X-centred hole sheets are more sensitive to the disorder than the outer electron jungle-gym and the hole ovoids between the Γ- and R-points. However, the smearing is not large, with well-defined darker regions showing negligible variation, and even the lighter regions indicate a smearing of less than 3% of the Brillouin zone. It should be emphasised that this characterisation of the smearing is likely to be sensitive to the supercell size and stoichiometry.

### Non-stoichiometric electronic structure determined from Compton scattering

**Figure 2:**
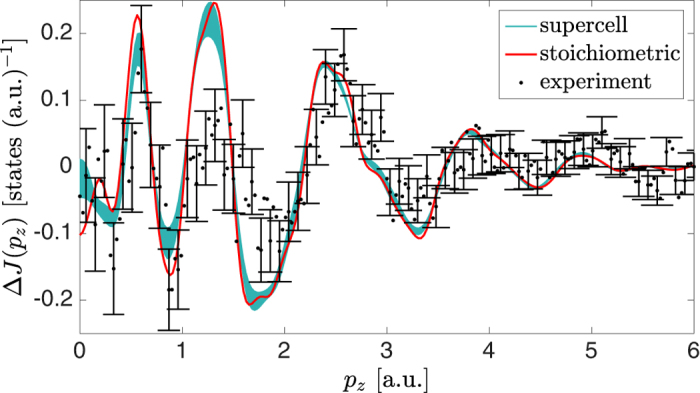
Comparison between the experimental and calculated Compton profiles. Difference, Δ*J*(*p*_*z*_), between Compton profiles with scattering vectors along the [110] and [100] directions for the experimental profiles (black dots), and those of the stoichiometric (red line) and supercell configurational average (blue line) calculations. The thickness of the blue line indicates the variation amongst the different supercell configurations and is two standard deviations wide. All of the calculations have been convoluted with a one-dimensional Gaussian with a full-width-at-half-maximum of 0.12 a.u. to approximate the experimental resolution. For clarity, the error bars are only plotted for every third data point and indicate statistical errors of one standard deviation.

A Compton profile, *J*(*p*_*z*_), is a one dimensional projection (double integral) of the underlying electron momentum density, thus containing information about the occupied momentum states and therefore about the Fermi surface^29^. Six Compton profiles were measured along equally spaced directions between the crystallographic [100] and [110] directions in the (001) plane (see *Methods*). The greatest directional difference, Δ*J*(*p*_*z*_), is observed in the difference between the [100] and [110] directions (Fig. 2), this being the largest angle between measured directions. Also shown are the calculated differences between profiles for stoichiometric MgCNi_3_, and those resulting from the configurational average of the supercell calculations. These calculated directional differences show reasonable agreement with the experimental differences, with peaks and troughs in the same places. Very rarely, the Fermi surface contribution to the directional differences is readily visible in the data (see, for example, Billingto﻿n *et al*.^36^,). However, our calculations confirm that the Fermi surface contribution to the Compton profile anisotropy for stoichiometric MgCNi_3_ is small, the anisotropy being dominated by the fully occupied bands. The stoichiometric and supercell calculations are very similar to one another, indicating that the effect of disorder is subtle in such directional differences. However, the size of the anisotropy in both calculations is distinctly overestimated compared to experiment (particularly the peaks and troughs in the momentum range 1.2 to 3.2 a.u.). This type of discrepancy is common in electronic structure calculations of the electron momentum density, and could arise due to limitations in the treatment of electron correlations in the local density approximation (LDA)^37^, although only anisotropic correlation effects would be visible in the directional differences. The overestimation of the anisotropy could also arise from self-interaction effects due to the LDA treatment of the Ni *d*-electrons, as is the case for Cu^38^.

**Figure 3:**
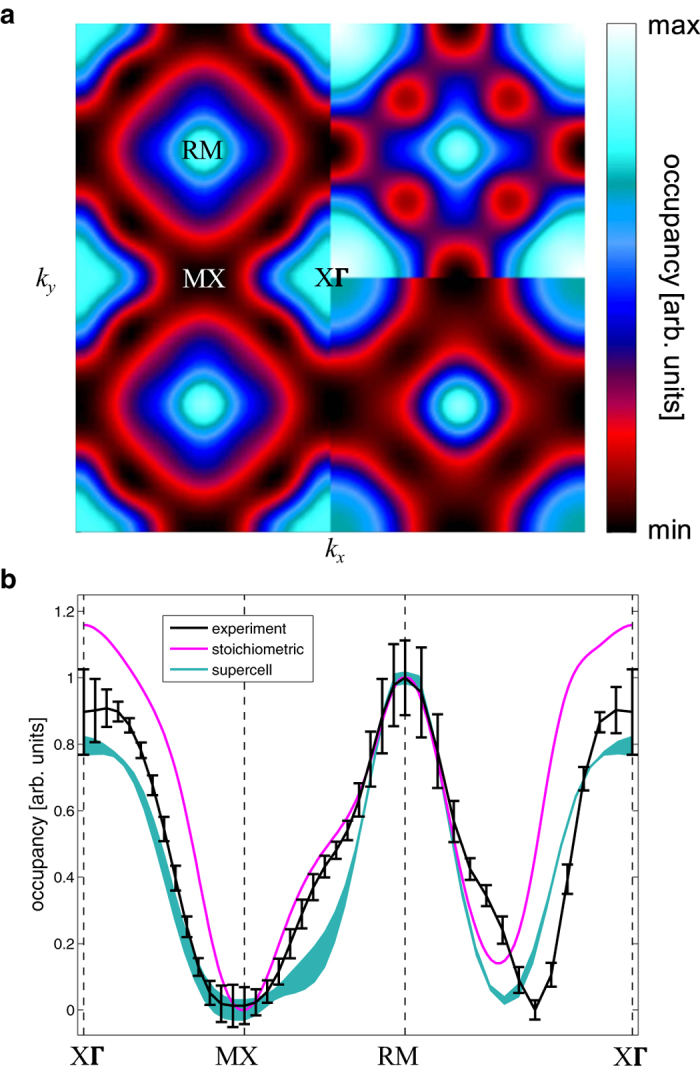
Comparison between the reconstructed experimental and calculated projected occupation densities. (**a**), Experimental occupation density (left half), and theoretical ***k***-space occupation densities, projected down [001], for the stoichiometric (top right), and supercell configurational average (bottom right) calculations. The plot is centred at the projected XΓ-point, and spans two Brillouin zones. The calculated projected occupation densities have been convoluted with a two-dimensional Gaussian approximating the experimental resolution function. The projected high-symmetry points are labelled. (**b**), Cuts through the [001]-projected experimental and theoretical occupation densities along certain (projected) high-symmetry directions. The thickness of the line for the supercell configurational average corresponds to two standard deviations and the experimental error bars indicate statistical errors of one standard deviation. All distributions have been normalised to unity at the projected RM-point.

A reconstruction from the 1D Compton profiles to a 2D projection was performed, and the resulting distribution was reduced to the first Brillouin zone, following the Lock-Crisp-West prescription^39^ (see *Methods*) such that the occupation density, *n*(**k**), projected down the [001] direction was obtained. Note that, with a sufficiently large number of Compton profiles measured along judiciously chosen directions, it is possible to reconstruct the three dimensional momentum density and thus the three dimensional Fermi surface^40^, however, with the small single crystal sample used in this study (see *Methods*) and a limited period of beamtime, such an approach was not feasible. Figure 3a shows the experimental and calculated two-dimensional occupation density, projected down [001]. Referring first of all to the stoichiometric calculation (top-right quadrant), the light blue circular regions around the RM-points originate from the projection of the band 2 jungle-gym arms which run the entire length of the Brillouin zone. The X-centred hole shells (band 1) can be discerned at the projected MX point, and the hole ovoids associated with the same band can be seen between XΓ and RM. Finally, the structure around XΓ, which varies from blue to white, originates from the band 2 distorted octahedron, and its appearance is strongly influenced by the projection of overlapping projected Fermi surfaces (in particular the hole ovoids). The experimental occupation density (left) is reproduced rather well by the configurationally averaged supercell (bottom right). Our KKR-CPA calculations of the projected ***k***-space occupation density at the supercell composition (not shown, for clarity) and configurational average supercell predictions of smearing upon the Γ-centred sheet correctly describe the experimental occupation density at the projected XΓ-point. Moreover, the supercell prediction of a slight thickening of the X-centred hole shells towards the Γ-point provides a much better representation of the size of the dip in the experimental occupation density around the projected MX-point. Between the XΓ- and RM-points, the position of the dip in the supercell result (caused by the hole ovoids) is slightly closer to the position of the dip in the experiment than the stoichiometric result, but does not agree completely with experiment, signifying some slight change in the positions of the crossings of the bands along the Γ-R direction, or of some additional smearing that is not reproduced in a supercell of this size. Figure 3b shows cuts through the [001]-projected experimental and calculated occupation densities, that essentially make clear the observations from the two-dimensional distributions in Fig. 3a.

Our experimental measurement of the Fermi surface of MgC_0.93_Ni_2.85_ reveals both hole and electron sheets of a similar shape to that predicted by LDA calculations for stoichiometric MgCNi_3_. The presence of C and Ni vacancies makes some parts of the Fermi surface sheet become rather smeared, but the other regions seem essentially unchanged. There is some evidence that the ovoid hole sheets between the Γ- and R-points are closer to the Γ-point than predicted, but a slight change in their shape (or smearing) could also potentially account for this discrepancy. That the supercell calculations are not completely able to describe the occupation density throughout the whole Brillouin zone is perhaps not so surprising given the small size of the supercell. It might also be important to consider the relaxation of the atomic positions in the presence of the vacancies, as has been revealed by powder neutron diffraction^26^, but this is beyond the scope of this primarily experimental study.

## Conclusions

We have presented a thorough investigation of the electronic structure and Fermi surface of MgC_0.93_Ni_2.85_, through high-resolution x-ray Compton scattering experiments combined with electronic structure calculations that treat the C and Ni vacancies. Supercell calculations indicate that the electronic structure, whilst smeared by disorder, does not drastically change in the presence of vacancies, and confirms the significant reduction in height of the vHs DOS peak, previously observed from photoemission experiments in polycrystalline samples with C vacancies^25^. This reduction in the DOS at the Fermi energy naturally provides an explanation for both the reduction in *T*_*c*_, and reduced ferromagnetic spin fluctuations observed in single crystals (which have more C and Ni vacancies than polycrystals). Our supercell configurational average (at a composition MgC_0.875_Ni_2.875_, which is close to our sample) indicates a disorder-induced smearing of the stoichiometric Fermi surface.

High-resolution x-ray Compton scattering measurements of the electron momentum density were performed, from which the projected ***k***-space occupation density was obtained. We find good agreement between the experimental occupation density and that predicted by our configurationally averaged supercell calculation, establishing the presence of both the hole and electron Fermi surface sheets predicted in calculations and confirming their shapes. Whilst none of our calculations completely describe the smearing of the occupation density in the presence of vacancies, we are able to identify significant smearing of the Γ-centred electron sheet, and slightly less smearing of the X-centred hole sheets; other parts of the Fermi surface appear to remain sharply defined.

With regards to the superconductivity, the most significant effect of the disorder is the suppression of the vHs and the reduction in the Fermi level DOS. If the pairing mechanism is purely electron-phonon, the BCS theory of superconductivity gives *T*_*c*_ ∝ exp(−1/*N*(*E*_F_)*V*), where *V* is the electron-phonon interaction potential^41^. Given that the experimental superconducting critical temperature extrapolates to a maximum of ${T}_{c}^{\max }=8.2$ K at perfect stoichiometry^26^, and assuming that *V* remains constant, this implies that the reduced *N*(*E*_F_) of the non-stoichiometric sample would give *T*_*c*_ ≈ 2.4 K. Since the experimental superconducting ${T}_{c}^{\exp }\approx 6.5$ K is not suppressed as strongly, this implies that the pair-breaking effect of spin fluctuations must also be reduced. Finally, we have unequivocally established that the Fermi surface of MgC_0.93_Ni_2.85_ is qualitatively similar to that predicted for the stoichiometric compound, and can conclude that the extensive previous theoretical investigations into the properties of MgCNi_3_ (that do not treat disorder) are based upon an experimentally justified description of its electronic structure.

## Methods

### Crystal growth

Single crystals of MgCNi_3_ were grown using a self-flux method within a high-pressure cubic anvil cell as described in ref. 21. A mixture of Mg, C, and Ni powders with a molar ratio of 1:1:3 were placed within a boron nitride crucible, and placed under a pressure of 3 GPa at room temperature. The assembly was then heated at constant pressure to above 1600 °C for one hour, before being slowly cooled to room temperature. The resultant growth lump was crushed, and single crystal samples were mechanically extracted.

The samples were characterised by magnetic susceptibility experiments and were found to have a superconducting transition temperature of *T*_*c*_ ≈ 6.5 K, with a narrow spread, presumably due to small variations in Ni and C content throughout the batch. The observed *T*_*c*_ agrees with previous measurements; it is slightly lower than the *T*_*c*_ = 6.7 K observed for MgCNi_2.8_ single crystals grown at higher pressures^35^, and lower than the *T*_*c*_ = 7.3 K observed in almost stoichiometric polycrystals^26^. The stoichiometry of the samples was determined by x-ray diffraction (XRD) experiments upon one of the small samples in the batch. The stoichiometry was found to be MgC_0.93_Ni_2.85_ (with an error of ±0.01 for Mg and C, and ±0.03 for Ni), with a lattice constant of *a* = 3.8008(2) Å at *T* = 100 K. These results are consistent with the stoichiometry of MgC_0.92_Ni_2.88_ reported for other single crystals grown by the same method, but our lattice constant differs from their value of *a* = 3.7913 Å^21^. This indicates a relatively large variation in lattice constant for samples grown by the same method, with effectively the same stoichiometry, and suggests a potentially significant uncertainty in lattice constant for different samples throughout the growth batch used in this study. Interestingly, the transition temperature is higher than the *T*_*c*_ ≈ 4.5 K expected for polycrystals with the C_0.93_ site occupation and a fully occupied Ni site^26^. The single crystal used in the Compton scattering experiment had the dimensions of approximately 1.0 × 0.5 × 0.4 mm^3^.

### Computational details

First-principles electronic structure calculations of the stoichiometric and non-stoichiometric compounds were performed. The highly-accurate full-potential Elk code^42^ with an augmented plane-wave plus local orbital basis was used for the majority of the calculations. Within the Elk code, virtual crystal approximation (VCA)^34^ and supercell calculations were employed to simulate the effect of vacancies on the electronic structure. As supercell calculations explicitly include any effects due to the local crystal structure in the vicinity of vacancies, they are expected (especially in the limit of infinite supercell size) to give more accurate predictions than the VCA. However, the supercell method is limited by the much larger computation time required to perform calculations. This is because the cells have to be large enough that the results are not dominated by vacancy–vacancy interactions, and because several calculations with vacancies placed in different locations need to be performed to correctly describe the random positioning of vacancies in a material. Furthermore, small supercells also restrict the available compositions.

For stoichiometric MgCNi_3_, calculations were performed with a cut-off for plane-waves in the interstitial region defined by |***G*** + ***k***|_max_ = 8.5/*R*_MT_, where *R*_MT_ is the average muffin tin radius. Convergence was achieved on a 32 × 32 × 32 ***k***-point mesh resulting in 969 ***k***-points in the irreducible Brillouin zone. The VCA calculations used the same parameters as the stoichiometric calculation, except the C and Ni atoms were replaced with effective atoms with nuclear charges of 5.58 and 26.63, respectively, thereby simulating the XRD stoichiometry. For the 2 × 2 × 2 supercell calculations, one C and one Ni atom were removed (thereby giving an effective stoichiometry of MgC_0.875_Ni_2.875_), and a cut-off for plane-waves in the interstitial region determined by |***G*** + ***k***|_max_ = 7.0/*R*_MT_ was used, with an 8 × 8 × 8 ***k***-point mesh resulting in 120 ***k***-points in the irreducible supercell Brillouin zone, corresponding to the same effective ***k***-point density as the stoichiometric and VCA calculations. For all of the Elk calculations, the muffin tin radii were set to 2.20 a.u., 1.10 a.u., and 2.40 a.u., for Mg, C, and Ni, respectively, and the Perdew-Wang/Ceperly-Alder local density approximation (PWCA-LDA)^43^ was used for the exchange-correlation functional. For consistency, the ambient temperature perovskite structure (space group $Pm\bar{3}m$), with Wyckoff positions of Mg at the 1*a* site (0, 0, 0), C at 1*b* (0.5, 0.5, 0.5), and Ni at the 3*c* site (0.5, 0.5, 0), and the experimental lattice constant for the nearly stoichiometric polycrystals of 3.81 Å was used for all calculations. This is because the experimental lattice constants at some of the compositions simulated in the calculations are unknown, and any estimations would likely contain a large uncertainty. Tests performed for the non-stoichiometric calculations at different lattice constants, including our XRD value, indicated negligible changes to the electronic structure compared to those introduced by the varying stoichiometry.

For the 2 × 2 × 2 supercells used in this study, there are three distinct configurations for the removal of a single C and Ni atom that are not related by symmetry or translation. The characteristic quantity of these configurations is the vacancy–vacancy distance in the periodic supercells. In the supercells, a vacancy separation of 4.25 Å appears twice as often by symmetry and translation as those with separations of 1.90 Å, and 5.70 Å. Test calculations were performed for different configurations with the same vacancy separation and these showed the same DOS (to within integration errors). The effect of relaxing the supercell crystal structure with vacancies was investigated for one configuration. This relaxation was found to have an almost negligible effect on the DOS compared to the effect of the vacancies themselves, whilst greatly increasing computation time due to the lowered symmetry and resulting increase in the number of ***k***-points in the irreducible Brillouin zone. As such, the unrelaxed crystal structure was used for all supercell calculations. Therefore, supercells were constructed for each of the three characteristic vacancy–vacancy distances, and all three were found to present qualitatively similar results. The weighted configurational average of all of the supercell calculations was taken, and this average was used for comparisons with the stoichiometric calculations, the non-stoichiometric calculations by other methods, and the experimental Compton scattering data.

It is worth noting that the Fermi surface of the supercell calculations cannot straightforwardly be obtained in a way comparable to the stoichiometric and VCA calculations because the crystal lattice has been artificially extended by including the extra cells. As a result, the band structure is folded into the supercell Brillouin zone which is much smaller than that of the stoichiometric and VCA calculations. However, it is possible to calculate the electron momentum distribution of the supercell calculations, and fold this distribution into the original Brillouin zone in order to obtain the ***k***-space occupation density. The supercell Fermi surface can then be determined from the configurationally averaged occupation density in the original Brillouin zone in the same manner that an experimental Fermi surface is obtained from a Compton scattering experiment. In order to make comparisons with our Compton scattering measurements, and to determine the Fermi surface from our supercell calculations, electron momentum densities and Compton profiles were calculated from the calculated electronic structure by the method of Ernsting *et al*.^44^.

Calculations were also performed using the Korringa-Kohn-Rostoker (KKR) method with the coherent potential approximation (CPA) that can effectively treat disorder within a mean field theory^45^.

### Compton scattering

The Compton scattering experiments were performed on beamline BL08W of the SPring-8 synchrotron, Japan. The high-resolution x-ray Compton spectrometer^46^ was used with an incident x-ray energy of 115 keV and a scattering angle of 165°. The spectrometer resolution could be described by a Gaussian with a full-width-at-half-maximum of 0.12 a.u. The measurements were made at room temperature (*T* = 298 K), and each profile had approximately 10^5^ counts in the Compton peak. Corrections were made for absorption, analyser and detector efficiencies, scattering cross-section, double scattering contributions and background. The core electron contributions, for the composition MgC_0.93_Ni_2.85_ determined by XRD, were then subtracted from each profile.

Compton scattering is a uniquely powerful probe of the ground-state electronic wave function^47, 48^. Since only occupied momentum states contribute to the momentum distribution *ρ*(***p***), it can be used for Fermi surface studies. A Compton profile, *J*(*p*_*z*_), is a double integral of the electron momentum distribution, *ρ*(***p***),

1 $$J({p}_{z})=\iint \rho ({\bf{p}})\,{\rm{d}}{p}_{x}{\rm{d}}{p}_{y},$$

where *p*_*z*_ is taken along the scattering vector and *ρ*(***p***) can be expressed as,

2 $$\rho ({\bf{p}})=\sum _{{\bf{k}},j}{n}_{{\bf{k}},j}{|\int {\psi }_{{\bf{k}},j}({\bf{r}}){{\rm{e}}}^{-{\rm{i}}{\bf{p}}\cdot {\bf{r}}}{\rm{d}}{\bf{r}}|}^{2},$$

where *ψ*_***k***,j_(***r***) is the wave function of the electron in band *j* with wave-vector ***k***, and *n*_***k***,*j*_ is its occupation.

A set of six Compton profiles were measured with scattering vectors equally spaced between the Γ-X and Γ-M directions of the simple cubic Brillouin zone. Tomographic reconstruction was used to obtain a once-integrated momentum distribution (a two-dimensional projection) in the plane of the scattering vectors. Cormack’s method was employed to perform the reconstruction^49^. The Lock-Crisp-West prescription^39^ was then applied to fold the projected ***p***-space distribution back into the first Brillouin zone in order to give the projected ***k***-space occupation density. The Fermi surface, which separates occupied from unoccupied states in ***k***-space, is manifest as a sharp change in the ***k***-space occupation density.

### Availability of Materials and Data

The underlying research materials can be accessed at the following DOI:10.5523/bris.ulryo0ap77x11zzatcwcgcu5q.

## References

[1] He T (2001). Superconductivity in the non-oxide perovskite MgCNi_3_. Nature.

[2] Ishida K (1998). Spin-triplet superconductivity in Sr_2_RuO_4_ identified by ^17^O knight shift. Nature.

[3] Norman MR, Pépin C (2003). The electronic nature of high temperature cuprate superconductors. Reports on Progress in Physics.

[4] Klimczuk T, Cava RJ (2004). Carbon isotope effect in superconducting MgCNi_3_. Phys. Rev. B.

[5] Rosner H, Weht R, Johannes MD, Pickett WE, Tosatti E (2001). Superconductivity near ferromagnetism in MgCNi_3_. Phys. Rev. Lett..

[6] Prozorov R, Snezhko A, He T, Cava RJ (2003). Evidence for unconventional superconductivity in the nonoxide perovskite MgCNi_3_ from penetration depth measurements. Phys. Rev. B.

[7] Young DP, Moldovan M, Adams PW (2004). Scaling behavior of the critical current density in MgCNi_3_ microfibers. Phys. Rev. B.

[8] Voelker, K. & Sigrist, M. Unconventional Superconductivity in MgCNi_3_. *Preprint at* https://arxiv.org/abs/cond-mat/0208367 (2002).

[9] Lin J-Y (2003). BCS-like superconductivity in MgCNi_3_. Phys. Rev. B.

[10] Shan L (2003). Influence of carbon concentration on the superconductivity in MgC_*x*_Ni_3_. Phys. Rev. B.

[11] Shan L (2003). *s*-wave pairing in MgCNi_3_ revealed by point contact tunneling. Phys. Rev. B.

[12] Ignatov AY, Savrasov SY, Tyson TA (2003). Superconductivity near the vibrational-mode instability in MgCNi_3_. Phys. Rev. B.

[13] Wälte A (2004). Evidence for strong electron-phonon coupling in MgCNi_3_. Phys. Rev. B.

[14] Singer PM, Imai T, He T, Hayward MA, Cava RJ (2001). ^13^C NMR investigation of the superconductor MgCNi_3_ up to 800 K. Phys. Rev. Lett..

[15] Mao ZQ (2003). Experimental determination of superconducting parameters for the intermetallic perovskite superconductor MgCNi_3_. Phys. Rev. B.

[16] Dugdale SB, Jarlborg T (2001). Electronic structure, magnetism, and superconductivity of MgC_*x*_Ni_3_. Phys. Rev. B.

[17] Singh DJ, Mazin II (2001). Superconductivity and electronic structure of perovskite MgCNi_3_. Phys. Rev. B.

[18] Diener P (2009). *s*-wave superconductivity probed by measuring magnetic penetration depth and lower critical field of MgCNi_3_ single crystals. Phys. Rev. B.

[19] Pribulová Z (2011). Superconducting energy gap in MgCNi_3_ single crystals: Point-contact spectroscopy and specific-heat measurements. Phys. Rev. B.

[20] Szczesniak R, Durajski A, Herok L (2015). Thermodynamic properties of antiperovskite MgCNi_3_ in superconducting phase. Solid State Communications.

[21] Gordon RT, Zhigadlo ND, Weyeneth S, Katrych S, Prozorov R (2013). Conventional superconductivity and hysteretic campbell penetration depth in single crystals MgCNi_3_. Phys. Rev. B.

[22] Shan L, Liu ZY, Ren ZA, Che GC, Wen HH (2005). Competition between BCS superconductivity and ferromagnetic spin fluctuations in MgCNi_3_. Phys. Rev. B.

[23] Daams JM, Mitrović B, Carbotte JP (1981). Simulation of the effects of paramagnons on a superconductor by a simple rescaling. Phys. Rev. Lett..

[24] Shein IR (2002). Effect of Co doping on the electronic structure of MgCNi_3_. Phys. Rev. B.

[25] Kim JH (2002). Photoemission and x-ray absorption study of MgC_1−*x*_Ni_3_. Phys. Rev. B.

[26] Amos T, Huang Q, Lynn J, He T, Cava R (2002). Carbon concentration dependence of the superconducting transition temperature and structure of MgC_*x*_Ni_3_. Solid State Communications.

[27] Shim JH, Kwon SK, Min BI (2001). Electronic structures of antiperovskite superconductors MgXNi_3_ (X = B, C, and N). Phys. Rev. B.

[28] Dugdale SB (2016). Life on the edge: a beginnerâ€™s guide to the Fermi surface. Physica Scripta.

[29] Dugdale SB (2014). Probing the Fermi surface by positron annihilation and Compton scattering. Low Temperature Physics.

[30] Szajek A (2001). Electronic structure of superconducting non-oxide perovskite MgCNi_3_. Journal of Physics: Condensed Matter.

[31] Li SY (2001). Normal state resistivity, upper critical field, and Hall effect in superconducting perovskite MgCNi_3_. Phys. Rev. B.

[32] Kumary TG (2002). Normal and superconducting states of MgCNi_3_ upon Fe and Co substitution and external pressure. Phys. Rev. B.

[33] Li SY (2002). Thermopower and thermal conductivity of superconducting perovskite MgCNi_3_. Phys. Rev. B.

[34] Nordheim L (1931). Zur elektronentheorie der metalle. i. Annalen der Physik.

[35] Lee H-S (2007). Growth of single crystals of MgCNi_3_. Advanced Materials.

[36] Billington D (2015). Magnetic frustration, short-range correlations and the role of the paramagnetic Fermi surface of PdCrO_2_. Scientific Reports.

[37] Shiotani N (1993). Compton scattering study of electron momentum density in vanadium. Journal of the Physical Society of Japan.

[38] Kubo Y, Sakurai Y, Shiotani N (1999). Effects of self-interaction correction on momentum density in copper. Journal of Physics: Condensed Matter.

[39] Lock DG, Crisp VHC, West RN (1973). Positron annihilation and Fermi surface studies: a new approach. Journal of Physics F: Metal Physics.

[40] Dugdale SB (2006). Observation of a strongly nested Fermi surface in the shape-memory alloy Ni_0.62_Al_0.38_. Phys. Rev. Lett..

[41] Bardeen J, Cooper LN, Schrieffer JR (1957). Theory of superconductivity. Phys. Rev..

[42] The Elk FP-LAPW code. http://elk.sourceforge.net.

[43] Perdew JP, Wang Y (1992). Accurate and simple analytic representation of the electron-gas correlation energy. Phys. Rev. B.

[44] Ernsting D (2014). Calculating electron momentum densities and Compton profiles using the linear tetrahedron method. Journal of Physics: Condensed Matter.

[45] Huhne T, Zecha C, Ebert H, Dederichs PH, Zeller R (1998). Full-potential spin-polarized relativistic Korringa-Kohn-Rostoker method implemented and applied to bcc Fe, fcc Co, and fcc Ni. Phys. Rev. B.

[46] Hiraoka N (2001). A new x-ray spectrometer for high-resolution Compton profile measurements at SPring-8. Journal of Synchrotron Radiation.

[47] Cooper MJ (1985). Compton scattering and electron momentum determination. Reports on Progress in Physics.

[48] Bansil A, Barbiellini B, Kaprzyk S, Mijnarends P (2001). Electron momentum density and Compton profile in disordered alloys. Journal of Physics and Chemistry of Solids.

[49] Kontrym-Sznajd G (1990). Three-dimensional image reconstruction with application in positron annihilation. Physica Status Solidi A.

